# Supplementary material for: NF-κB epigenetic attractor landscape drives breast cancer heterogeneity
Source: NPJ Syst Biol Appl. 2025 Nov 24;11:135. doi: 10.1038/s41540-025-00611-0 (PMC12644762; doi:10.1038/s41540-025-00611-0)
Supplement: Supplementary file 1 — Supplementary materials [file 41540_2025_611_MOESM1_ESM.pdf]

Supplementary Materials for

Title: NF-κB Epigenetic Attractor Landscape Drives Breast Cancer Heterogeneity

Francisco Lopes*[1,2], Bruno R. B. Pires[3,4], Alexandre A. B. Lima[1], Renata Binato[4], Eliana Abdelhay[4]

[1]Universidade Federal do Rio de Janeiro (UFRJ), Campus Duque de Caxias Professor Geraldo Cidade; Duque de Caxias, Brazil.

[2]Institute for Quantitative and Computational Biosciences (QCBio), University of California Los Angeles (UCLA); Los Angeles, USA.

[3]Departamento de Biofísica e Biometria (DBB), Universidade Estadual do Rio de Janeiro (UERJ); Rio de Janeiro, Brazil.

[4]Stem Cell Laboratory, Instituto Nacional de Câncer; Rio de Janeiro, Brazil.
*Corresponding author: flopes@ufrj.br

This PDF file includes:

Supplementary Figures 1 to 4
Supplementary Table 1
Legends for Supplementary Movies 1 and 2.

	Model Reactions					
	**Description**	**Reaction**	**Forward constants**	**Forward constants units**	**Backward constants**	**Backward constants units**
**1**	NFkB dimer formation	p50 + p65 ↔ NFkB	2.66319E-06	pl^2/(min*#)	1.69683E+00	pl/min
**2**	NFkB binds p65 promoter	NFkB + N0p65 ↔ N1p65	8.90887E-04	pl^2/(min*#)	1.04883E+02	pl/min
**3**	p65 RNA synthesis	N1p65 → N1p65 + RNAp65	5.62307E+01	pl/min		
**4**	p65 synthesis	RNAp65 → RNAp65 + p65	4.495E+01	pl/min		
**5**	NFkB binds p50 promoter	NFkB + N0p50 ↔ N1p50	9.02865E-01	pl^2/(min*#)	8.2909E+04	pl/min
**6**	p50 RNA synthesis	N1p50 → N1p50 + RNAp50	3.70696E+01	pl/min		
**7**	p50 synthesis	RNAp50 → RNAp50 + p50	9.38E+00	pl/min		
**8**	NFkB binds SIP1 promoter	NFkB + N0SIP1 ↔ N1SIP1	8.90887E-04	pl^2/(min*#)	2.5257E+00	pl/min
**9**	SIP1 RNA synthesis	N1SIP1 → N1SIP1 + RNASIP1	7.30761E+02	pl/min		
**10**	SIP1 synthesis	RNASIP1 → RNASIP1 + SIP1	1.1684E+02	pl/min		
**11**	NFkB binds SLUG promoter	NFkB + N0SLUG ↔ N1SLUG	8.90887E-04	pl^2/(min*#)	7.925E+01	pl/min
**12**	SLUG RNA synthesis	N1SLUG → N1SLUG + RNASLUG	2.62313E+03	pl/min		
**13**	SLUG synthesis	RNASLUG → RNASLUG + SLUG	1.1684E+02	pl/min		
**14**	NFkB binds TWIST promoter	NFkB + N0TWIST1 ↔ N1TWIST1	8.90887E-04	pl^2/(min*#)	4.85495E+00	pl/min
**15**	TWIST RNA synthesis	N1TWIST1 → N1TWIST1 + RNATWIST1	8.08799E+03	pl/min		
**16**	TWIST synthesis	RNATWIST1 → RNATWIST1 + TWIST1	1.1684E+02	pl/min		
**17**	p50 RNA constitutive syntesis	0 → RNAp50	6.12305E+00	#/min		
**18**	p65 RNA constitutive syntesis	0 → RNAp65	9.19739E+00	#/min		
**19**	p50 RNA degradation	RNAp50 → 0	1.015E-01	pl/min		
**20**	p65 RNA degradation	RNAp65 → 0	1.07848E-01	pl/min		
**21**	SIP1 RNA degradation	RNASIP1 → 0	1.01208E-01	pl/min		
**22**	SLUG RNA degradation	RNASLUG → 0	1.01208E-01	pl/min		
**23**	TWIST1 RNA degradation	RNATWIST1 → 0	1.01208E-01	pl/min		
**24**	p65 degradation	p65 → 0	5.2506E-02	pl/min		
**25**	p50 degradation	p50 → 0	3.01325E-02	pl/min		
**26**	SIP1 degradation	SIP1 → 0	4.83112E-02	pl/min		
**27**	SLUG degradation	SLUG → 0	4.83112E-02	pl/min		
**28**	TWIST1 degradation	TWIST1 → 0	4.83112E-02	pl/min		

Supplementary Figure 1. Gene Regulatory Reactions and Parameters. Blue parameters were calculated based on experimentally determined mRNA and protein half-lives, and transcription and translation rate constants (43). Green values were derived from the average corresponding values for a mammalian cell.

$$
\begin{aligned}
\frac{d([\text{NFkB}])}{dt} = &+(k_{1_{\text{"NFkB dimer formation"}}} \cdot [p50] \cdot [p65] \\
&-k_{2_{\text{"NFkB dimer formation"}}} \cdot [\text{NFkB}]) \\
&-(k_{1_{\text{"NFkB binds p50 gene promoter"}}} \cdot [\text{NFkB}] \cdot [\text{NO}p50] \\
&-k_{2_{\text{"NFkB binds p50 gene promoter"}}} \cdot [\text{N1}p50]) \\
&-(k_{1_{\text{"NFkB binds p65 gene promoter"}}} \cdot [\text{NFkB}] \cdot [\text{NO}p65] \\
&-k_{2_{\text{"NFkB binds p65 gene promoter"}}} \cdot [\text{N1}p65]) \\
&-(k_{1_{\text{"NFkB binds SIP1 gene promoter"}}} \cdot [\text{NFkB}] \cdot [\text{NO}SIP1] \\
&-k_{2_{\text{"NFkB binds SIP1 gene promoter"}}} \cdot [\text{N1}SIP1]) \\
&-(k_{1_{\text{"NFkB binds SLUG gene promoter"}}} \cdot [\text{NFkB}] \cdot [\text{NO}SLUG] \\
&-k_{2_{\text{"NFkB binds SLUG gene promoter"}}} \cdot [\text{N1}SLUG]) \\
&-(k_{1_{\text{"NFkB binds TWIST gene promoter"}}} \cdot [\text{NFkB}] \cdot [\text{NO}TWIST1] \\
&-k_{2_{\text{"NFkB binds TWIST gene promoter"}}} \cdot [\text{N1}TWIST1])
\end{aligned} \tag{1}
$$

$$
\begin{aligned}
\frac{d([\text{SLUG}])}{dt} = &-k_{1_{\text{"SLUG degradation"}}} \cdot [\text{SLUG}] \\
&+k_{1_{\text{"SLUG synthesis"}}} \cdot [\text{RNASLUG}]
\end{aligned} \tag{2}
$$

$$
\begin{aligned}
\frac{d([\text{TWIST1}])}{dt} = &+k_{1_{\text{"TWIST synthesis"}}} \cdot [\text{RNATWIST1}] \\
&-k_{1_{\text{"TWIST1 degradation"}}} \cdot [\text{TWIST1}]
\end{aligned} \tag{3}
$$

$$
\begin{aligned}
\frac{d([\text{RNATWIST1}])}{dt} = &-k_{1_{\text{"TWIST1 RNA degradation"}}} \cdot [\text{RNATWIST1}] \\
&+k_{1_{\text{"TWIST RNA synthesis"}}} \cdot [\text{N1TWIST1}]
\end{aligned} \tag{4}
$$

$$
\begin{aligned}
\frac{d([\text{RNASIP1}])}{dt} = &-k_{1_{\text{"SIP1 RNA degradation"}}} \cdot [\text{RNASIP1}] \\
&+k_{1_{\text{"SIP1 RNA synthesis"}}} \cdot [\text{N1SIP1}]
\end{aligned} \tag{5}
$$

$$
\begin{aligned}
\frac{d([\text{RNASLUG}])}{dt} = &-k_{1_{\text{"SLUG RNA degradation"}}} \cdot [\text{RNASLUG}] \\
&+k_{1_{\text{"SLUG RNA synthesis"}}} \cdot [\text{N1SLUG}]
\end{aligned} \tag{6}
$$

$$
\begin{aligned}
\frac{d([\text{p65}])}{dt} = &-k_{1_{\text{"p65 degradation"}}} \cdot [\text{p65}] \\
&-k_{1_{\text{"NFkB dimer formation"}}} \cdot [p50] \cdot [p65] \\
&+k_{2_{\text{"NFkB dimer formation"}}} \cdot [\text{NFkB}] \\
&+k_{1_{\text{"p65 synthesis"}}} \cdot [\text{RNAp65}]
\end{aligned} \tag{7}
$$

Supplementary Figure 2. Model differential equations. System of Ordinary Differential Equations obtained by applying the Law of Mass Action to the model reactions in **Supplementary Figure 1**. Continue…

$$\frac{d([\mathrm{RNAp65}])}{dt} = +k_{1_{\text{"p65 RNA synthesis"}}} \cdot [\mathrm{N1p65}] - k_{1_{\text{"p65 RNA degradation"}}} \cdot [\mathrm{RNAp65}] + \left(k_{1_{\text{"p65 RNA constitutive synthesis"}}}\right) \tag{8}$$

$$\frac{d([\mathrm{RNAp50}])}{dt} = +k_{1_{\text{"p50 RNA synthesis"}}} \cdot [\mathrm{N1p50}] - k_{1_{\text{"p50 RNA degradation"}}} \cdot [\mathrm{RNAp50}] + \left(k_{1_{\text{"p50 RNA constitutive synthesis"}}}\right) \tag{9}$$

$$\frac{d([\mathrm{p50}])}{dt} = -\left(k_{1_{\text{"NFkB dimer formation"}}} \cdot [p50] \cdot [p65] - k_{2_{\text{"NFkB dimer formation"}}} \cdot [\mathrm{NFkB}]\right) - k_{1_{\text{"p50 degradation"}}} \cdot [p50] + k_{1_{\text{"p50 synthesis"}}} \cdot [\mathrm{RNAp50}] \tag{10}$$

$$\frac{d([\mathrm{NOSLUG}])}{dt} = -\left(k_{1_{\text{"NFkB binds SLUG gene promoter"}}} \cdot [\mathrm{NFkB}] \cdot [\mathrm{NOSLUG}] - k_{2_{\text{"NFkB binds SLUG gene promoter"}}} \cdot [\mathrm{N1SLUG}]\right) \tag{11}$$

$$\frac{d([\mathrm{NOSIP1}])}{dt} = -\left(k_{1_{\text{"NFkB binds SIP1 gene promoter"}}} \cdot [\mathrm{NFkB}] \cdot [\mathrm{NOSIP1}] - k_{2_{\text{"NFkB binds SIP1 gene promoter"}}} \cdot [\mathrm{N1SIP1}]\right) \tag{12}$$

$$\frac{d([\mathrm{SIP1}])}{dt} = -k_{1_{\text{"SIP1 degradation"}}} \cdot [\mathrm{SIP1}] + k_{1_{\text{"SIP1 synthesis"}}} \cdot [\mathrm{RNASIP1}] \tag{13}$$

$$\frac{d([\mathrm{NOTWIST1}])}{dt} = -\left(k_{1_{\text{"NFkB binds TWIST gene promoter"}}} \cdot [\mathrm{NFkB}] \cdot [\mathrm{NOTWIST1}] - k_{2_{\text{"NFkB binds TWIST gene promoter"}}} \cdot [\mathrm{N1TWIST1}]\right) \tag{14}$$

$$\frac{d([\mathrm{N1TWIST1}])}{dt} = +k_{1_{\text{"NFkB binds TWIST gene promoter"}}} \cdot [\mathrm{NFkB}] \cdot [\mathrm{NOTWIST1}] - k_{2_{\text{"NFkB binds TWIST gene promoter"}}} \cdot [\mathrm{N1TWIST1}] \tag{15}$$

$$\frac{d([\mathrm{N1SLUG}])}{dt} = +k_{1_{\text{"NFkB binds SLUG gene promoter"}}} \cdot [\mathrm{NFkB}] \cdot [\mathrm{NOSLUG}] - k_{2_{\text{"NFkB binds SLUG gene promoter"}}} \cdot [\mathrm{N1SLUG}] \tag{16}$$

$$\frac{d([\mathrm{N1SIP1}])}{dt} = +k_{1_{\text{"NFkB binds SIP1 gene promoter"}}} \cdot [\mathrm{NFkB}] \cdot [\mathrm{NOSIP1}] - k_{2_{\text{"NFkB binds SIP1 gene promoter"}}} \cdot [\mathrm{N1SIP1}] \tag{17}$$

$$\frac{d([\mathrm{N1p65}])}{dt} = +k_{1_{\text{"NFkB binds p65 gene promoter"}}} \cdot [\mathrm{NFkB}] \cdot [\mathrm{NOp65}] - k_{2_{\text{"NFkB binds p65 gene promoter"}}} \cdot [\mathrm{N1p65}] \tag{18}$$

$$\frac{d([\mathrm{N1p50}])}{dt} = +k_{1_{\text{"NFkB binds p50 gene promoter"}}} \cdot [\mathrm{NFkB}] \cdot [\mathrm{NOp50}] - k_{2_{\text{"NFkB binds p50 gene promoter"}}} \cdot [\mathrm{N1p50}] \tag{19}$$

$$\frac{d([\mathrm{NOp65}])}{dt} = -\left(k_{1_{\text{"NFkB binds p65 gene promoter"}}} \cdot [\mathrm{NFkB}] \cdot [\mathrm{NOp65}] - k_{2_{\text{"NFkB binds p65 gene promoter"}}} \cdot [\mathrm{N1p65}]\right) \tag{20}$$

$$\frac{d([\mathrm{NOp50}])}{dt} = -\left(k_{1_{\text{"NFkB binds p50 gene promoter"}}} \cdot [\mathrm{NFkB}] \cdot [\mathrm{NOp50}] - k_{2_{\text{"NFkB binds p50 gene promoter"}}} \cdot [\mathrm{N1p50}]\right) \tag{21}$$

Supplementary Figure 2. Model differential equations. System of Ordinary Differential Equations obtained by applying the Law of Mass Action to the model reactions in **Supplementary Figure 1**. *End.*

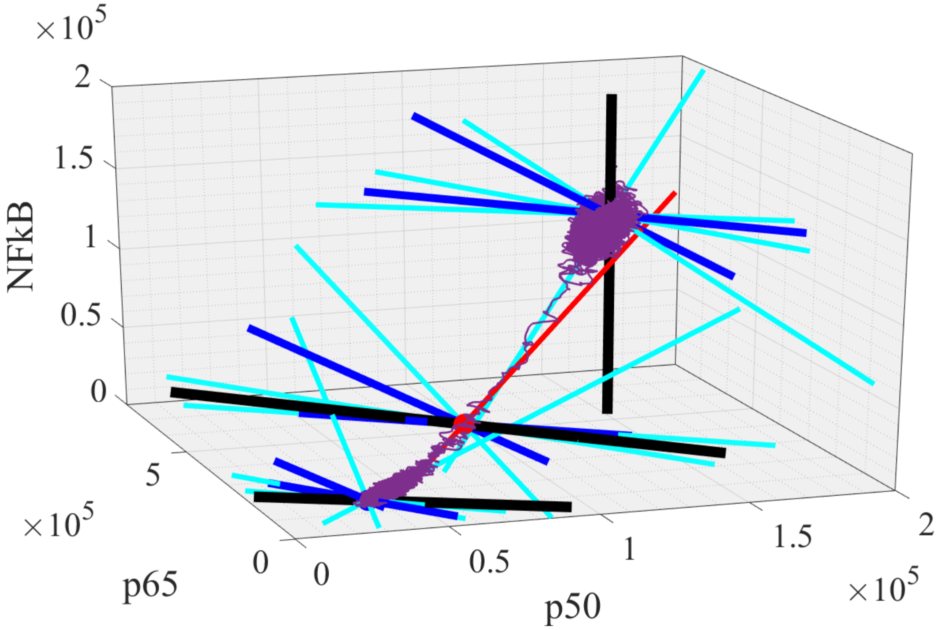

Supplementary Figure 3. Complete set of eigenvectors for the model. Projection of all seven eigenvectors with nonzero eigenvalues, determined from each stationary state, within the p65-p50-NFκB phase-space. The magnitude of the associated eigenvalues varies widely, ranging from –2.0E+5 to 2.5E-3. To deal with this wide range, we classified the eigenvectors into three categories based on their eigenvalues: light blue for weak (1.0E-3 to 1.0E-1), dark blue for medium (1.0 to 1.0E+2), and black for strong (1.0E+4 or higher). The only eigenvector with a positive eigenvalue is the red one, which is classified as weak (2.5E-3). The trajectory from a single-cell stochastic simulation is shown in purple.

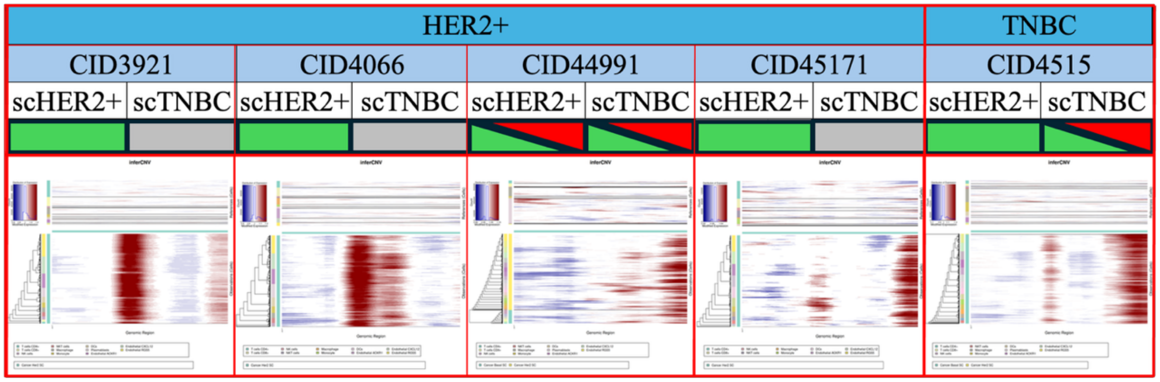

Supplementary Figure 4. TNBC cells inside HER2+ and TNBC bulk-classified samples exhibit HER2 gene copy gains. TNBC cells inside HER2+ and TNBC bulk-classified samples exhibit HER2 gene copy gains. Top panel: The first row indicates bulk classification. The second row shows individual patient samples (CID3921, CID4066, CID44991, CID45171, CID4515). The third row represents single-cell classifications within each sample. Green and red boxes denote copy number gains and losses, respectively, inferred using inferCNV, while gray indicates the absence of the corresponding single-cell subtype. Bottom panel: Heatmap illustrating relative gene expression intensities along chromosome 17, generated using inferCNV from scRNA-seq (9).

Supplementary Table 1. mRNA and protein copy number estimates. mRNA copy number was estimated by multiplying the number of molecules in a healthy cell (43) by the relative increase given by the qPCR data. Protein copy number was estimated from the RNA copy number, the mRNA translation rate constant, and the protein degradation rate (43). TWIST1, SLUG, and SIP1 mRNA molecules were estimated from the median of all values in a healthy cell. The same approach was used for their mRNA translation rate constant and protein degradation rate. NFκB1 (p50) was estimated from the RelA (p65) qPCR data.

	HER2 cell				TNBC cell			
	qPCR		mRNA copy number	Protein copy number	qPCR		mRNA copy number	Protein copy number
	Mean	Standard Error			Mean	Standard Error		
RelA	3.33E0	3.94E-1	1.35E+2	1.16E+5	1.54E+1	6.84E+1	6.25E+2	5.35E+5
NF-κB1			1.04E+2	3.25E+4			4.83E+2	1.50E+5
TWIST1	4.78E+3	6.26E+2	8.30E+4	2.01E+8	8.81E+3	2.78E+2	1.53E+5	3.71E+8
SLUG	1.85E+2	5.30E+1	3.22E+3	7.79E+6	1.75E+3	4.35E+1	3.04E+4	7.36E+7
SIP1	5.61E+2	4.04E0	9.75E+3	2.36E+7	8.12E+2	1.63E+2	1.41E+4	3.42E+7

Supplementary Movie 1. Spontaneous transition from HER2+ to TNBC subtypes. The purple line indicates the full trajectory in the p50-p65 phase space. The time-lapse animation shows fluctuations within each attractor basin as well as the spontaneous transition.

Supplementary Movie 2. The unstable intermediate state provides a fluctuation-susceptible slow route along the weak eigenvectors. The time-lapse animation shows that the protein copy number fluctuate along the weak eigenvectors in the p50-p65 phase space. The full trajectory is depicted in Figure 4A.
